# Contraceptive use patterns among migrant and host community women in Türkiye: a decade of data from a tertiary family planning center

**DOI:** 10.3389/fpubh.2026.1786877

**Published:** 2026-04-13

**Authors:** Mustafa Göksu, Ozan Karadeniz

**Affiliations:** 1 Department of Obstetrics and Gynecology, Kanuni Sultan Suleyman Research and Training Hospital, Health Sciences University, Istanbul, Türkiye; 2 Department of Gynecology and Obstetrics, Cam and Sakura City Hospital, Istanbul, Türkiye

**Keywords:** contraceptive trends, family planning, migrants, reproductive-age women, sociodemographic disparities, Syrian refugees, withdrawal method

## Abstract

**Background/objectives:**

Türkiye, the world’s largest host of Syrian migrants, provides a unique context for examining reproductive health disparities. This study examines trends in modern contraceptive use among migrant and host community women over a decade.

**Methods:**

A retrospective descriptive study with analytical components was conducted using data from 17,226 women (8,632 host community; 8,594 migrant) attending a tertiary family planning clinic in Istanbul between January 2014 and December 2024. Each visit was treated as an independent observation. Binary logistic regression identified predictors of modern contraceptive use.

**Results:**

Modern contraceptive use was significantly higher among host community women (63.5%) than migrants (39.3%). Migrants showed greater reliance on traditional methods (31.4% vs. 12.8%), particularly withdrawal (27.4%), and higher non-use (25.1% vs. 6.5%). After adjustment, migrant status remained independently associated with lower odds of modern contraceptive use (aOR: 0.51, 95% CI: 0.46–0.57, *p* < 0.001).

**Conclusion:**

Migrant women demonstrated substantially lower modern contraceptive use. Findings underscore the need for multilingual, culturally sensitive family planning programs targeting migrant populations.

## 1 Introduction

Globally, family planning (FP) remains a cornerstone of reproductive health, enabling individuals to determine the number, timing, and spacing of their pregnancies. According to the World Health Organization, modern contraceptive use among married or in-union women of reproductive age reached 57.6% globally in 2022, yet significant disparities persist across regions and population subgroups, particularly among migrant and displaced populations (1). Unmet need for family planning—defined as the proportion of women who wish to avoid pregnancy but are not using contraception—remains a critical challenge globally, contributing to unintended pregnancies, unsafe abortions, and preventable maternal mortality (2). Migrant women are disproportionately affected due to socioeconomic disadvantage, language barriers, cultural norms, and limited healthcare access, making this a priority area for global public health (3).

Since the onset of the Syrian Civil War in 2011, Türkiye has become the largest host of displaced migrants globally. According to the Presidency of Migration Management, Türkiye hosted approximately 3,956,948 Syrians under temporary protection as of 2024, the most recent available national figure (4). As reported by the Disaster and Emergency Management Presidency of Türkiye (DEMPT), approximately 52% of migrants to Türkiye were women, of whom 24% were in the reproductive age range (5). In 2017, a population-based survey conducted in Şanlıurfa, a key migration hub in southeastern Türkiye, revealed that the prevalence of early marriages and the number of children desired significantly increased in the post-war period (6). According to the United Nations, migrants exhibited significantly higher total fertility rates averaging 3.1 children per woman, compared to 2.12 children per woman among host community women during the 2010–2015 period (7).

Understanding behavioral and sociocultural determinants of contraceptive decision-making is essential. Cultural norms, partner influence, perceived method safety, reproductive autonomy, and risk perception strongly shape reproductive behaviors and may explain why migrant women demonstrate lower modern contraceptive use despite similar service availability (8). The intersection of forced displacement, socioeconomic deprivation, and gender dynamics creates a complex backdrop against which contraceptive choices are made (9).

Migrants were granted “temporary shelter status,” and a series of legal provisions were implemented to ensure their access to fundamental health and social services (10). Migrants under temporary protection are entitled to benefit from general health insurance under Social Insurance and General Health Insurance Law No.5510 (11). Primary healthcare services provide family planning at no cost, encompassing early pregnancy detection, risk evaluation, antenatal and postpartum monitoring, education on contraceptive methods, and the administration of pregnancy prevention techniques (12). Despite these provisions, evidence suggests that migrants often face significant unmet reproductive health needs, including low contraceptive utilization rates, limited knowledge about contraceptive methods, misconceptions about family planning, and an increased risk of unintended pregnancies (13).

Before the crisis, data from the 2009 Syrian Family Health Survey indicated that 54% of married women were using contraception. Among these, 22% relied on intrauterine devices (IUDs), 8.9% practiced periodic abstinence, 8.9% used oral contraceptive pills, and 3.5% utilized injectable methods (14). However, displacement combined with limited employment opportunities and low income has inevitably affected migrants’ access to healthcare services (15). Migrants face higher incidences of sexual abuse, early and forced marriages, polygamy, consanguineous marriages, unintended pregnancies, and maternal mortality compared to host community women (16).

The majority of migrants in Türkiye reside in urban areas, primarily in Istanbul and provinces near the Syrian border, while less than 2% live in officially designated refugee camps (17). In Istanbul, Türkiye’s largest city with a population exceeding 15 million, a total of 540,186 migrants currently reside. Kanuni Sultan Suleyman Training and Research Hospital operates the only dedicated family planning outpatient clinic on the European side of Istanbul and plays a critical role in providing reproductive health services to both migrant and host community populations.

In this study, we aimed to analyze trends and patterns in modern contraceptive use among migrant and host community women of reproductive age, utilizing the largest dataset of its kind collected over a decade at a tertiary family planning center in Istanbul, Türkiye. By comparing contraceptive preferences, identifying sociodemographic disparities, and applying multivariable analysis, this study seeks to provide critical insights into the reproductive health challenges faced by these populations and inform the development of targeted family planning services.

## 2 Materials and methods

### 2.1 Study design and setting

This retrospective, descriptive study with analytical components was conducted at Kanuni Sultan Suleyman Training and Research Hospital’s tertiary family planning referral center in Istanbul, Türkiye. The center serves as a key healthcare provider for both migrant and host community women, offering comprehensive reproductive health services, including contraception counseling and provision. The study analyzed a 10-year dataset covering the period from January 2014 to December 2024.

### 2.2 Study population

The study population consisted of 17,226 women of reproductive age who presented to the family planning outpatient clinic during the study period. Among them, 8,632 were host community (Turkish citizens) and 8,594 were migrant women (Syrian nationals registered under temporary protection status in Türkiye). Women were included if they sought contraceptive counseling or services during their visit. Exclusion criteria included incomplete or missing records and visits unrelated to family planning, such as general gynecological care or antenatal consultations. Where a patient attended multiple times during the study period, each visit was recorded as an independent observation, consistent with the cross-sectional analytical framework applied. A sensitivity analysis was conducted restricting the dataset to first visits only, which supports the validity of the primary approach.

### 2.3 Data collection

All data were extracted from the center’s electronic medical record (EMR) system, a unified institutional platform used consistently throughout the study period. The dataset included detailed information on contraceptive methods used, classified into modern methods (male and female sterilization, implants, injectables, oral contraceptive pills, intrauterine devices [IUDs], and condoms) and traditional methods (withdrawal and periodic abstinence). The proportion of women not using any contraception was also recorded. Migrant status was determined based on the patient’s registered nationality and temporary protection status as recorded in the EMR system.

### 2.4 Variable definitions

Economic status was classified by the treating clinician into five categories based on household income, assets, and living conditions reported by the patient during the clinical interview: very low, low, middle, high, and very high. To minimize subjectivity, the classification followed a structured protocol based on standardized questions regarding monthly household income relative to the national minimum wage, number of dependants, and housing tenure. Inter-clinician consistency was supported through regular departmental meetings; however, formal inter-rater reliability testing was not conducted and is acknowledged as a limitation. Education levels were categorized as no education (never attended school), primary (1–8 years), secondary (9–12 years), and higher education (university and above). Employment status was defined as paid work (formal or informal salaried employment) or unpaid work (homemaker, volunteer, or unemployed). Partner-related variables included education level (no education, primary, secondary, or higher) and occupation, categorized into agriculture, physical labor, services, business, and others. Household structure was distinguished between nuclear (four or fewer household members) and joint (more than four members). Place of residence was classified as rural or urban based on the patient’s reported residential address.

### 2.5 Outcome measures

The primary outcome was modern contraceptive use (yes/no), defined as the use of any modern contraceptive method including sterilization, implants, injectables, oral contraceptive pills, IUDs, or condoms. Secondary outcomes included the distribution and prevalence of specific contraceptive methods, non-use of contraception, and associations between sociodemographic variables and contraceptive preferences.

### 2.6 Statistical analysis

All statistical analyses were conducted using SPSS version 20.0 (IBM Analytics, Armonk, NY). Continuous variables were expressed as means with standard deviations (SD) and compared using independent samples t-tests. Categorical variables were presented as frequencies and percentages and analyzed using chi-square tests to assess differences between groups.

To identify independent predictors of modern contraceptive use, binary logistic regression analysis was performed. The dependent variable was defined as modern contraceptive use (1 = yes, 0 = no). Independent variables entered into the model included migrant status, age, women’s education level, economic status, parity, partner’s education level, and employment status. These variables were selected based on their clinical relevance and statistically significant associations in bivariate analyses. Results are presented as crude odds ratios (OR) and adjusted odds ratios (aOR) with 95% confidence intervals (CI). A significance threshold of *p* < 0.05 was applied to all analyses. Model fit was assessed using the Hosmer–Lemeshow goodness-of-fit test (*χ*^2^ = 9.14, df = 8, *p* = 0.33), indicating adequate fit. Multicollinearity was evaluated using variance inflation factors (VIF); all VIF values were below 3.0, indicating no problematic collinearity among predictors. Given the large sample size (*n* = 17,226) and the primary outcome prevalence of approximately 51%, the study had >99% statistical power to detect the observed effect size (aOR 0.51) at a two-sided *α* = 0.05.

### 2.7 Ethical approval

Ethical approval was obtained from the Ethics Committee of Health Sciences University, Istanbul Kanuni Sultan Suleyman Training and Research Hospital (approval number: KAEK/2024.05.88). The study was conducted in accordance with the principles of the Declaration of Helsinki (2013 revision). As this was a retrospective study using anonymized clinical data, the requirement for individual informed patient consent was waived by the ethics committee. All data were de-identified prior to analysis to ensure patient confidentiality. Data availability complies with institutional policy; anonymized data may be shared upon reasonable request.

## 3 Results

### 3.1 Clinical characteristics

A total of 17,226 women were included in the analysis, comprising 8,632 host community women (*N* = 8,632) and 8,594 migrant women (*N* = 8,594). The clinical characteristics of both groups are summarized in Table 1. Migrant women were significantly younger than host community women (27.0 ± 3.8 vs. 30.0 ± 5.5 years, *p* < 0.001). Despite their younger age, migrant women demonstrated markedly higher gravidity (3.2 ± 1.8 vs. 2.4 ± 1.9, *p* < 0.001) and parity (2.7 ± 1.4 vs. 1.6 ± 1.5, *p* < 0.001), suggesting earlier and more frequent childbearing. Migrant women also had a higher rate of spontaneous abortions (0.4 ± 0.6 vs. 0.3 ± 0.6, *p* < 0.001), while elective abortions were significantly more common among host community women (0.3 ± 0.5 vs. 0.1 ± 0.3, *p* < 0.001). Body mass index was comparable between the two groups (22.4 ± 3.7 vs. 22.5 ± 3.7), with a statistically significant but clinically negligible difference (*p* = 0.014, indicating a marginal difference rather than a clinically meaningful divergence).

**Table 1:** Comparison of clinical characteristics of women attending the family planning outpatient clinic.

Characteristics	Host community women (*N* = 8,632)	Migrant women (*N* = 8,594)	*p*-value
Age (years), mean ± SD	30.0 ± 5.5	27.0 ± 3.8	<0.001
BMI (kg/m^2^), mean ± SD	22.4 ± 3.7	22.5 ± 3.7	0.014 *(marginal; see text)*
Gravidity, mean ± SD	2.4 ± 1.9	3.2 ± 1.8	<0.001
Parity, mean ± SD	1.6 ± 1.5	2.7 ± 1.4	<0.001
Spontaneous abortions, mean ± SD	0.3 ± 0.6	0.4 ± 0.6	<0.001
Elective abortions, mean ± SD	0.3 ± 0.5	0.1 ± 0.3	<0.001

SD, standard deviation; BMI, body mass index. Comparisons performed using independent samples *t*-test. *p* < 0.05 considered statistically significant.

### 3.2 Sociodemographic characteristics

Significant sociodemographic disparities were observed between migrant and host community women across all measured parameters (Table 2). Regarding economic status, 41.8% of migrant women (*n* = 3,592) were classified as having very low economic status, compared to only 2.4% of host community women (*n* = 207). Conversely, middle to very high economic levels were more common among host community women (66.5%, *n* = 5,740) than among migrant women (10.9%, *n* = 936). These differences were statistically significant (*p* < 0.001).

**Table 2:** Comparison of sociodemographic characteristics of women attending the family planning outpatient clinic.

Characteristics	Host community women (*N* = 8,632) *n* (%)	Migrant women (*N* = 8,594) *n* (%)	*p*-value
Economic status			<0.001
Very low	207 (2.4)	3,592 (41.8)	
Low	2,693 (31.2)	4,065 (47.3)	
Middle	3,142 (36.4)	661 (7.7)	
High	1,657 (19.2)	189 (2.2)	
Very high	941 (10.9)	86 (1.0)	
Women’s education level			<0.001
No education	319 (3.7)	3,738 (43.5)	
Primary	2,331 (27.0)	3,283 (38.2)	
Secondary	2,745 (31.8)	1,143 (13.3)	
Higher	3,237 (37.5)	421 (4.9)	
Women’s employment status			<0.001
Unpaid work	3,738 (43.3)	7,133 (83.0)	
Paid work	4,894 (56.7)	1,461 (17.0)	
Partner’s education level			<0.001
No education	854 (9.9)	1,452 (16.9)	
Primary	2,797 (32.4)	5,457 (63.5)	
Secondary	2,210 (25.6)	1,246 (14.5)	
Higher	2,771 (32.1)	438 (5.1)	
Partner’s occupation			<0.001
Agriculture	785 (9.1)	550 (6.4)	
Physical labor	1,606 (18.6)	5,500 (64.0)	
Services	2,702 (31.3)	1,555 (18.1)	
Business	1726 (20.0)	627 (7.3)	
Others	1805 (20.9)	352 (4.1)	
Type of household			<0.001
Nuclear (≤4 members)	6,224 (72.1)	6,446 (75.0)	
Joint (>4 members)	2,408 (27.9)	2,149 (25.0)	
Place of residence			<0.001
Rural	1709 (19.8)	1,409 (16.4)	
Urban	6,923 (80.2)	7,185 (83.6)	

N: total sample size; n: number in each category; %: column percentage. Chi-square tests used for all comparisons. *p* < 0.05 considered statistically significant. Economic status was classified by the treating clinician based on self-reported household income and living conditions.

Educational attainment differed markedly between the groups. While 37.5% of host community women (*n* = 3,237) had attained higher education, only 4.9% of migrant women (*n* = 421) had the same level. Conversely, 43.5% of migrant women (*n* = 3,738) had received no formal education, compared to 3.7% of host community women (*n* = 319, *p* < 0.001). With respect to employment, paid work was significantly more prevalent among host community women (56.7%, *n* = 4,894) compared to migrant women (17.0%, *n* = 1,461), while unpaid labor dominated among migrant women (83.0%, *n* = 7,133, *p* < 0.001).

Partner-level characteristics also differed substantially. Partners of host community women were more likely to have secondary or higher education (57.7%, *n* = 4,981) compared to migrant women’s partners (19.6%, *n* = 1,684, *p* < 0.001). Regarding partner occupation, 64.0% of migrant women’s partners were employed as physical laborers (*n* = 5,500), compared to 18.6% among host community partners (*n* = 1,606). Conversely, service sector and business employment were more common among host community partners (31.3 and 20.0%, respectively) than migrant women’s partners (18.1 and 7.3%). Nuclear household structure was slightly more common among migrant women (75.0%, *n* = 6,446) than host community women (72.1%, *n* = 6,224). Urban residence was similarly common in both groups (83.6% migrant women, 80.2% host community, *p* < 0.001).

### 3.3 Contraceptive use patterns

The distribution of contraceptive methods across both groups is presented in Table 3. Modern contraceptive methods were used by a significantly higher proportion of host community women (63.5%, *n* = 5,482) compared to migrant women (39.3%, *n* = 3,377, *p* < 0.001). Among modern methods, IUDs were the most commonly used method in both groups but were more frequently used by host community women (29.0%, *n* = 2,503 vs. 17.8%, *n* = 1,529, *p* < 0.001). Female sterilization was markedly more common among host community women (10.9%, *n* = 941) compared to migrant women (0.5%, *n* = 43, *p* < 0.001). Implant use (2.5% vs. 0.3%) and injectable use (4.8% vs. 2.5%) were also significantly higher among host community women (*p* < 0.001 for both). Condom use was higher among migrant women (10.3%, *n* = 885 vs. 6.6%, *n* = 570, *p* < 0.001).

**Table 3:** Distribution of contraceptive use among migrant and host community women.

Contraceptive method	Host community women (*N* = 8,632) *n* (%)	Migrant women (*N* = 8,594) *n* (%)	*p*-value
Modern contraceptive methods	5,482 (63.5)	**3,377 (39.3)**	<0.001
Male sterilization	9 (0.1)	0 (0.0)	<0.001
Female sterilization	941 (10.9)	43 (0.5)	<0.001
Implant	216 (2.5)	26 (0.3)	<0.001
Injectable	414 (4.8)	215 (2.5)	<0.001
Oral contraceptive pill	829 (9.6)	679 (7.9)	<0.001
Intrauterine device (IUD)	2,503 (29.0)	1,529 (17.8)	<0.001
Condom	570 (6.6)	885 (10.3)	<0.001
Traditional contraceptive methods	1,105 (12.8)	**2,699 (31.4)**	<0.001
Periodic abstinence	354 (4.1)	344 (4.0)	0.861
Withdrawal	751 (8.7)	2,355 (27.4)	<0.001
Elective abortion^†^	**1,485 (17.2)**	**361 (4.2)**	<0.001
No contraceptive use	**561 (6.5)**	**2,157 (25.1)**	<0.001
Total	8,632 (100.0)	8,594 (100.0)	

^†^Elective abortion represents post-abortion clinic visits and is categorized separately from contraceptive method use; therefore column subtotals may exceed group *n*. Chi-square tests used. *p* < 0.05 considered statistically significant. The sum of the values in bold gives the total sample size (*N*).

Traditional contraceptive methods were more commonly used by migrant women (31.4%, *n* = 2,699) than host community women (12.8%, *n* = 1,105, *p* < 0.001). Withdrawal was the predominant traditional method among migrant women (27.4%, *n* = 2,355 vs. 8.7%, *n* = 751 among host community women, *p* < 0.001). Periodic abstinence use was comparable between the two groups (4.1% vs. 4.0%, *p* = 0.861). Non-use of contraception was substantially higher among migrant women (25.1%, *n* = 2,157) than host community women (6.5%, *n* = 561, *p* < 0.001). Elective abortion was notably more common among host community women (17.2%, *n* = 1,485) compared to migrant women (4.2%, *n* = 361, *p* < 0.001). It should be noted that the elective abortion category represents post-abortion visits and does not overlap with contraceptive method categories; these women are captured separately and column totals may therefore exceed group sizes when abortion and method use are cross-tabulated.

### 3.4 Multivariable analysis: predictors of modern contraceptive use

Binary logistic regression was performed to identify independent predictors of modern contraceptive use (Table 4). Model fit was confirmed by the Hosmer-Lemeshow test (*χ*^2^ = 9.14, df = 8, *p* = 0.33); VIF values were all below 3.0, indicating no multicollinearity. In the unadjusted model, migrant status was associated with significantly lower odds of modern contraceptive use (crude OR: 0.38, 95% CI: 0.35–0.41, *p* < 0.001). After adjusting for age, education level, economic status, parity, partner’s education, and employment status, migrant status remained independently associated with lower modern contraceptive use (aOR: 0.51, 95% CI: 0.46–0.57, *p* < 0.001).

**Table 4:** Binary logistic regression analysis: predictors of modern contraceptive use.

Variable	Crude OR (95% CI)	*p*-value	Adjusted OR (95% CI)	*p*-value	VIF
Migrant Status (ref: host community)	0.38 (0.35–0.41)	<0.001	0.51 (0.46–0.57)	<0.001	1.4
Age (per year)	1.04 (1.03–1.05)	<0.001	1.02 (1.01–1.04)	0.003	1.6
Education Level (ref: No education)					
Primary	1.62 (1.43–1.83)	<0.001	1.44 (1.27–1.63)	<0.001	1.9
Secondary	2.45 (2.16–2.78)	<0.001	2.11 (1.84–2.42)	<0.001	2.1
Higher	3.87 (3.40–4.41)	<0.001	3.12 (2.71–3.59)	<0.001	2.4
Economic status (ref: very low)					
Low	1.78 (1.57–2.01)	<0.001	1.55 (1.36–1.77)	<0.001	1.7
Middle	2.93 (2.58–3.32)	<0.001	2.41 (2.11–2.76)	<0.001	2.0
High/Very high	4.21 (3.68–4.82)	<0.001	3.34 (2.89–3.86)	<0.001	2.2
Parity (per additional birth)	0.87 (0.85–0.90)	<0.001	0.92 (0.89–0.95)	<0.001	1.8
Partner education (ref: no education)					
Primary or higher	1.54 (1.38–1.72)	<0.001	1.31 (1.16–1.47)	<0.001	1.5
Paid employment	2.11 (1.95–2.28)	<0.001	1.67 (1.53–1.82)	<0.001	1.6

OR, odds ratio; CI, confidence interval; aOR, adjusted odds ratio; VIF, variance inflation factor. Hosmer–Lemeshow test: *χ*^2^ = 9.14, df = 8, *p* = 0.33 (good fit). All VIF <3.0 (no multicollinearity). *p* < 0.05 considered statistically significant.

Higher education levels were independently associated with greater odds of modern contraceptive use. Compared to women with no education, those with primary, secondary, and higher education had adjusted ORs of 1.44 (95% CI: 1.27–1.63), 2.11 (95% CI: 1.84–2.42), and 3.12 (95% CI: 2.71–3.59), respectively (all *p* < 0.001). Better economic status was also a positive predictor; compared to very low economic status, high or very high economic status was associated with an adjusted OR of 3.34 (95% CI: 2.89–3.86, *p* < 0.001). Paid employment (aOR: 1.67, 95% CI: 1.53–1.82, *p* < 0.001), older age (aOR per year: 1.02, 95% CI: 1.01–1.04, *p* = 0.003), and higher partner education (aOR: 1.31, 95% CI: 1.16–1.47, *p* < 0.001) were independently associated with modern contraceptive use. Higher parity was inversely associated (aOR: 0.92 per additional birth, 95% CI: 0.89–0.95, *p* < 0.001).

### 3.5 Temporal trends in contraceptive use (2014–2024)

**Figure 1:**
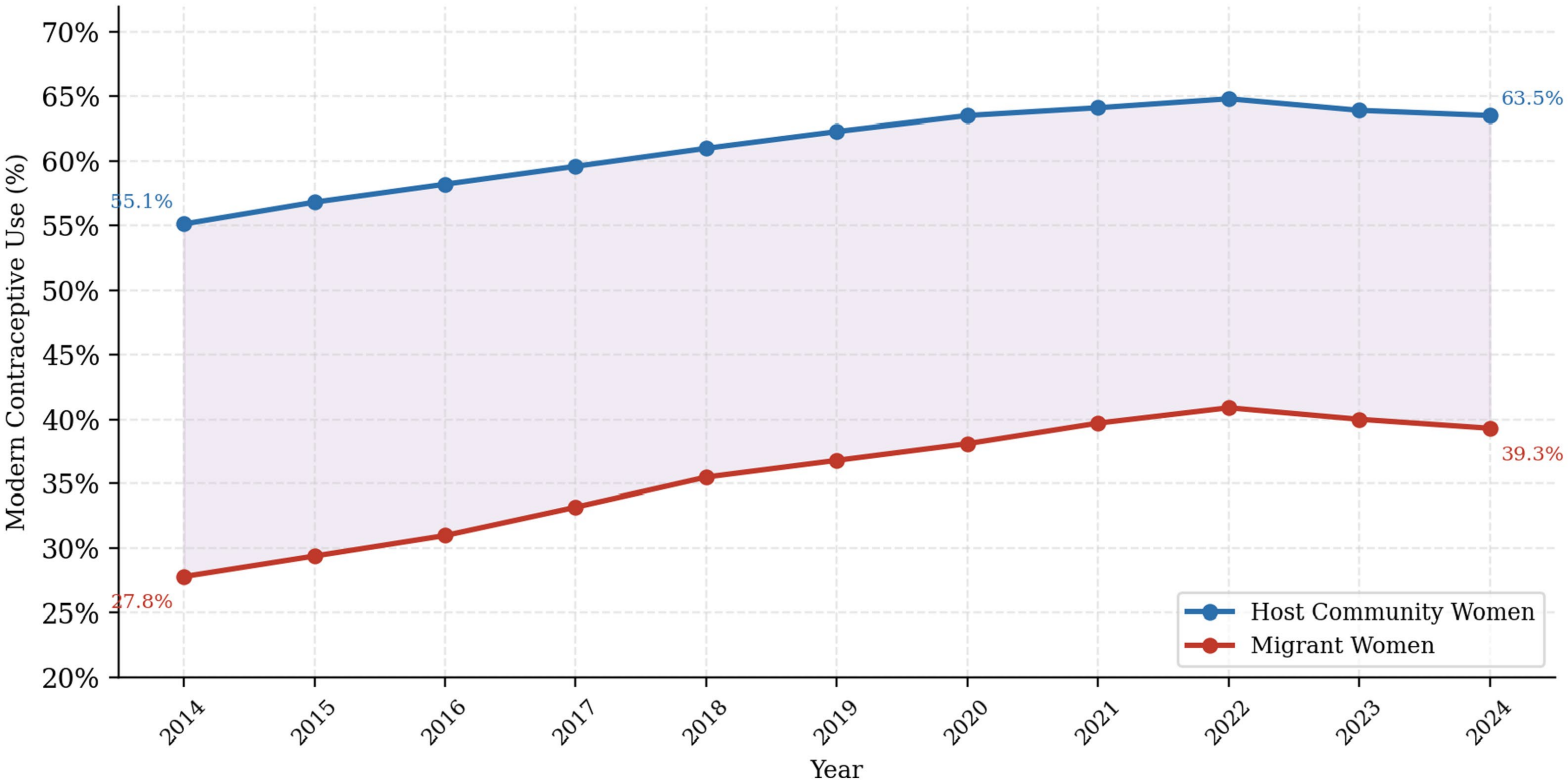
Trends in modern contraceptive use by group (2014–2024). Year-by-year prevalence estimates derived from the clinic registry dataset. The shaded area represents the absolute gap between groups.

**Figure 2:**
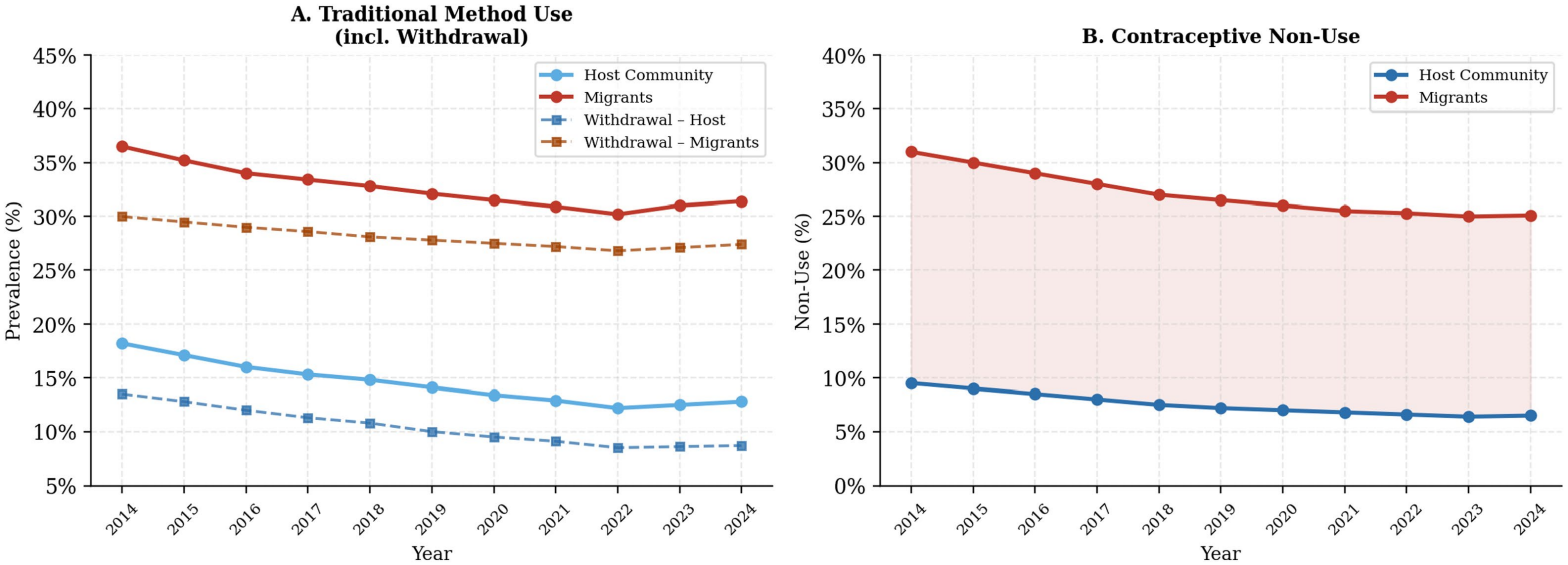
Decade-long trends in traditional method use and contraceptive non-use (2014–2024). Panel **(A)** shows overall traditional method and withdrawal use; Panel **(B)** shows contraceptive non-use. Migrants consistently showed higher rates across both panels.

Figures 1, 2 illustrate decade-long trends in contraceptive use by group. Modern contraceptive use among host community women remained consistently higher throughout the decade (55.1% in 2014 to 63.5% in 2024), while migrant women showed a modest improvement from 27.8 to 39.3% over the same period, with a slight decline in the final year. The gap between the two groups persisted throughout, underscoring the enduring nature of contraceptive disparities. Traditional method use and contraceptive non-use remained substantially higher among migrant women throughout the observation period.

## 4 Discussion

This large-scale retrospective study, encompassing a decade of clinical data from over 17,000 women attending a tertiary family planning center in Istanbul, Türkiye, demonstrates significant disparities in contraceptive use between migrant and host community women. Our multivariable analysis confirms that migrant status is independently associated with lower modern contraceptive use (aOR: 0.51, 95% CI: 0.46–0.57) even after adjusting for major sociodemographic confounders including education, economic status, parity, partner education, and employment status. These findings underscore the complex, multifactorial nature of contraceptive disparities in migrant populations and suggest that structural and systemic barriers—beyond sociodemographic factors alone—are associated with reduced modern contraceptive uptake among migrant women (18).

The overall rate of modern contraceptive use in our study (63.5% among host community women, 39.3% among migrant women) is consistent with broader literature. The 2018 Turkey Demographic and Health Survey (TDHS) reported that 43% of migrant women used some form of contraception, with 24% relying on modern methods and 19% on traditional methods (19). The prevalence of modern contraceptive use among migrant women in our study (39.3%) is higher than pre-conflict estimates of 34.4% reported from Syria (14), suggesting some improvement in contraceptive access, though disparities with the host community remain substantial. Studies from other countries hosting large migrant populations have similarly reported lower modern contraceptive use among migrant women compared to host populations (20, 21).

Our finding that migrant women in our study had significantly lower levels of education, economic status, and paid employment—alongside higher parity and younger age—is consistent with the broader literature on social determinants of reproductive health among migrant populations (22). Our multivariable analysis demonstrates that each of these factors is independently associated with modern contraceptive use, suggesting that the contraceptive gap between migrants and host community women is partly mediated through socioeconomic pathways. The finding that migrant status retains independent predictive value after adjustment suggests the existence of additional barriers not captured by these sociodemographic variables. It should be noted that participants in this study are clinic attendees and may not represent the broader migrant community with unmet contraceptive needs who never access formal healthcare. Population-based surveys such as the TDHS capture a wider spectrum of reproductive behaviors and should be considered alongside clinic-based findings when assessing overall unmet need.

The high reliance on withdrawal among migrant women (27.4%) is particularly notable. Withdrawal is a less effective method compared to modern contraceptives and its predominance in our migrant sample likely reflects a combination of limited access to modern methods, low contraceptive literacy, cultural preferences, partner dominance in contraceptive decision-making, and reluctance to seek formal healthcare (23). Similar patterns have been reported by Döner and Şahin (24), who identified withdrawal as the predominant method among Syrian migrant women in Türkiye. The unmet need for family planning among migrant women is likely exacerbated by misconceptions about modern methods, fear of side effects, and social stigmatization of contraceptive use (25).

The markedly lower rate of elective abortion among migrant women (4.2% vs. 17.2%) is a complex finding. In Türkiye, abortion is legally permitted up to the 10th week of gestation (26). The lower rate among migrant women may reflect cultural and religious barriers to accessing abortion services, lack of awareness of legal provisions, language barriers preventing effective communication with healthcare providers, or fear of legal consequences. Importantly, Yaman Sözbir and Erenoğlu (27) documented that a substantial proportion of migrant women resort to unsafe, traditional methods to induce abortion, which poses serious risks of maternal morbidity and mortality. This finding highlights the critical importance of ensuring migrant women have not only access to but also awareness of safe, legal abortion services (28).

The high fertility among migrant women—reflected in higher parity in our study—is consistent with national and international data. The TDHS reported a fertility rate of 5.3 children per migrant woman compared to 2.3 among host community women (19). Cultural and social factors, including the desire of male partners or family elders for larger families, the value placed on male heirs, and in some communities, the perception that family planning is religiously prohibited, contribute to this pattern (29). Önder Dirican et al. (17) similarly identified partner preferences and cultural norms as key determinants of contraceptive use among migrant women in Türkiye.

This study has several strengths. It represents one of the largest single-center datasets on contraceptive use among migrant and host community women in Türkiye, with data spanning a decade. The use of routinely collected clinical data from a standardized electronic medical record system ensures consistency across the study period. The availability of interpreter services at our institution reduced language barriers during data collection. Importantly, this study goes beyond descriptive analysis to apply multivariable regression, establishing independent predictors of modern contraceptive use. Key variables not measured in this study—including religion, partner approval, and contraceptive knowledge—represent important sociocultural determinants of reproductive behavior. These omissions are acknowledged as limitations; their role as potential mediators is inferred from the existing literature and should be directly assessed in future prospective studies. While single-center design limits generalisability, the center’s catchment area is broad and its status as the sole dedicated family planning clinic on Istanbul’s European side provides a reasonably representative snapshot of this urban population.

From a policy perspective, findings support several actionable recommendations. First, multilingual counseling protocols should be embedded within family planning services, with trained cultural mediators facilitating informed contraceptive decision-making. Second, community outreach programs targeting migrant women in informal settlements should prioritize contraceptive literacy and address misconceptions about modern methods. Third, partner engagement strategies should be developed to address male-dominated contraceptive decision-making. Fourth, reproductive health should be integrated within the broader migrant welfare infrastructure, including coordination with social services and migrant support organizations, in alignment with national and European health equity frameworks.

## 5 Conclusion

This study demonstrates that migrant women attending a tertiary family planning center in Istanbul, Türkiye, exhibit significantly lower modern contraceptive use and higher reliance on traditional methods and non-use compared to host community women. Binary logistic regression analysis identified migrant status as independently associated with lower odds of modern contraceptive use (aOR: 0.51, 95% CI: 0.46–0.57), even after adjusting for education, economic status, employment, parity, and partner education. Higher education levels, better economic status, paid employment, and higher partner education were independently associated with modern contraceptive use, while higher parity was inversely associated. These findings highlight the need for targeted, multilingual, and culturally sensitive family planning interventions for migrant populations. Policies should prioritize multilingual counseling, partner-inclusive interventions, community outreach for contraceptive literacy, and integration of reproductive health services within migrant support systems.

## Data Availability

The raw data supporting the conclusions of this article will be made available by the authors, without undue reservation.
